# Exploring the experiences and preferences of South Asian patients' of primary care in England since COVID‐19

**DOI:** 10.1111/hex.13982

**Published:** 2024-02-05

**Authors:** Nicola Small, Yumna Masood, Fiona Stevenson, Benjamin C. Brown, Caroline Sanders, Brian McMillan, Helen Atherton, Tandrima Mazumdar, Nigat Ara, Humera Haqqani, Sudeh Cheraghi‐Sohi

**Affiliations:** ^1^ Division of Population Health, Health Services Research and Primary Care, Centre for Primary Care and Health Services Research, Faculty of Biology, Medicine, and Health, School of Health Sciences University of Manchester Manchester UK; ^2^ Research Department of Primary Care and Population Health, Institute of Epidemiology and Health Care University College London London UK; ^3^ Unit of Academic Primary Care, Warwick Medical School University of Warwick Coventry UK; ^4^ Division of Psychology and Mental Health, Faculty of Biology, Medicine, and Health, School of Health Sciences University of Manchester Manchester UK

**Keywords:** COVID‐19 access, general practice, qualitative, South Asian, telephone, triage

## Abstract

**Introduction:**

Remote (digital and/or telephone) access and consultation models are being driven by national policy with the goal being that the National Health Service operate on a remote‐first (digital‐first) basis by 2029. Previous research has suggested that remote methods of access to care and consulting may act to widen health inequalities for certain patients and/or groups such as those from ethnic minorities. South Asian (SA) patients comprise the largest ethnic minority group in England. Understanding the experiences and needs of this group is critical to ensuring that general practice can deliver equitable, quality health care.

**Methods:**

Qualitative study. 37 participants (from Indian, Pakistani and/or Bangladeshi background) were recruited to take part in either in‐person preferred language focus groups or remote semistructured interviews in the English language. Thematic analysis was conducted to identify themes in the qualitative data.

**Findings:**

Three major interlinked themes were identified: (1) reduced access, (2) reduced patient choice and (3) quality and safety concerns. The findings highlight access issues split by (i) general issues with appointment access via any remote means and (ii) specific issues related to language barriers creating additional barriers to access and care. Some patients valued the convenience of remote access but also raised concerns regarding appointment availability and reduced patient choice. Face‐to‐face consultations were preferable but less available. The findings underscore how participants perceived remote care to be of lesser quality and less safe. Concerns were greatest for those with limited English proficiency (LEP), with the removal of non‐verbal aspects of communication and ‘hands‐on’ care leading to perceptions of reduced psycho‐social safety.

**Conclusion:**

SA patients' experiences of remote‐led primary care access and care delivery were negative with only a minority viewing it positively and for certain limited scenarios. Face‐to‐face models of care remain the preferred mode of consultation, particularly for those with LEP. Hybrid models of access offer patients the greatest choice, and are likely to meet the varying needs of the South‐Asian patient population going forwards. The remote first approach to primary care may be achievable as a service ideal, but its limitations need to be recognised and accounted for to ensure that primary care can be an equitable service, both now and in the future.

**Public Contribution:**

Members of the public were involved in all phases of research in the study. This included co‐working in partnership throughout the study including, reviewing patient‐facing documents, recruiting participants, data facilitation, translation work, interpretation of the data and co‐authors on this manuscript. The key to the success of our study was collaborative teamwork, which involved experienced members of the public with SA cultural knowledge working together with and integral to the research team for all components.

## 1 INTRODUCTION

Before COVID‐19, we might have been offered a telephone appointment some of the time, but most of the time, people were offered, and wanted a face‐to‐face appointment.

Since COVID‐19, most people's appointments have been over the telephone, with only a small number of people seeing their general practitioners (GPs) face‐to‐face in general practice surgery or via a video call.^1^ This was to ensure patient and general practice staff safety. During COVID‐19, ‘triage’ systems, in other words, when, how and who we are seen by were introduced. These became known as ‘Total triage’, with *all* patients being triaged remotely (via telephone or digital systems). This was a new way of working for practitioners but also for patients in terms of accessing care and it was all rapidly introduced due to the circumstances and nature of COVID‐19.^2^ Decisions as to who got an appointment, when and what type of appointment (face‐to‐face, telephone or video‐call) were made either by receptionists, nurse practitioners and/or GPs over the phone or alternatively by patients, or their carers, filling in a form online which a member of GP staff would then use to decide the need for and type of appointment that patient would get. This system essentially removed patient choice for appointment type, which is why it is called ‘total’ triage. The government of the United Kingdom recently (13 May 2021) announced a change to allow patients a choice again of what type of consultation they feel they need, including face‐to‐face. How patients and GP practices responded to this new announcement is a question we wanted to explore as part of this research.

Individual practices implemented modes of access, triage and consultation delivery differently and current models of access, triage and consultation still vary widely (telephone, face‐to‐face and/or digital forms) as individual practices choose their preferred ways of enabling access and care.^3^ Many patients have had prolonged exposure to these new ways of accessing and receiving care. It is therefore timely to understand patient perspectives and experiences of these various modes of access, triage and consultation modes to ascertain the impact of any such changes, particularly with respect to any unintended consequences.^4^ We know little of how specific patient groups experienced care since mandated total triage has been removed and research conducted pre‐pandemic has suggested that remote methods of access to care and consulting may act to widen health inequalities.^5^, ^6^ One such potentially affected group are those from ethnic minority backgrounds.

People from ethnic minority backgrounds in the United Kingdom often have worse health outcomes than the general population,^7^, ^8^ may have health disease profiles distinct from the rest of the population^9^ and they are at a greater risk of patient safety issues occurring versus the general population.^10^, ^11^ Reasons for this vary and include poorer access to care as highlighted by International studies.^12^ Contributing factors to this poorer access include patients' and/or carers language differences (not speaking the dominant language when accessing health care services), racism (perceived and enacted) and culture are recognised as significant predictors of access to care and subsequent health care delivery/outcomes.^13^, ^14^, ^15^, ^16^ A recent systematic review on online consultations also highlighted new, technology‐associated access inequity factors in that patients who speak the native language are more likely to use online consultations.^6^ Studies report multiple cultural and religious barriers common to all ethnic minority groups, which need to be considered when understanding low uptake to accessing services.^17^ Cultural values and beliefs have been found to vary in terms of the stigma and meanings associated with managing certain conditions, such as dementia. Although the prevalence of dementia is higher in some ethnic minorities, issues like stigma mean that they are also less likely to engage with dementia services.^17^, ^18^

According to the 2011 census, people from a South Asian (SA) background (India, Pakistan, Bangladesh, Sri Lanka) make up the largest proportion of the non‐White population in England and Wales, with those of Indian and Pakistani origin being the largest groups, with 1.4 million identifying with the Indian ethnic group (2.5%), and 1.1 million with the Pakistani ethnic group (2.0%).^19^ Understanding the experiences of the largest ethnic group in England when accessing and using primary care since COVID‐19 and the associated changes in the way primary care may be accessed and delivered is therefore important to ensure lessons are learned, particularly as we move towards a digital‐first primary care service.^20^ For example, issues such as digital poverty and low digital literacy have the potential to disproportionately affect older ethnic minority adults in particular.^21^ Digital literacy and particular cultural values e.g. health beliefs around mental health condition causes^22^ have also been shown to impact the preferences and experiences of this group in accessing care since COVID‐19.^23^ Other factors such as lower income, also disproportionately affect access to digital technologies and internet use amongst ethnic minorities^21^ and those from Bangladeshi or Pakistani backgrounds have the highest rates of income poverty in England.^24^ Such factors help create the digital divide and have the potential to become ‘super social determinants of health’ going forwards.^25^ Remote means of accessing and receiving care, also place an even greater emphasis on the need for good communication skills of practice staff *and* their patients to explain and understand patients' clinical need(s). Barriers to communication are not entirely overcome by the use of interpreters.^26^, ^27^ Interpersonal barriers to communication, which may result from language and/or cultural differences between a patient and their doctor, may however be reduced if the language spoken by a doctor and/or his or her ethnicity concords (i.e., matches) with the patients' characteristics.^28^ Although digital systems may in theory remove language and ethnicity concordance barriers by the use of automated translation services, practices may not choose to switch these on due to potential risks in automatic translation *creating* communication errors.^29^

General practice is the gateway to the broader National Health Service (NHS) in the United Kingdom and has a key role to play in the equitable, integrated, and responsive NHS envisioned by policymakers.^30^, ^31^ Understanding and responding to the experiences of different types of patients and/or patient groups' routes and experiences of accessing and using general practice since new models of access and care were implemented is key to realising this vision. This study aims to understand the experiences of SA patients in accessing and using general practice in the United Kingdom since COVID‐19 restrictions in accessing and receiving care were lifted.

## 2 METHODS

### 2.1 Design

Qualitative study using focus groups and semistructured interviews.

### 2.2 Patient and public involvement (PPI)

Three PPI multilingual public collaborators of SA origins (T. M., N. A., H. H.) were involved from the inception of the study (bid development) with two collaborators (T. M. and N. A.) actively involved in leading recruitment and data collection (topic guide co‐development and piloting, recruitment and focus group facilitation, transcription and translation work) as well as in the production of this manuscript. Of note, the topic guide was piloted during a group discussion with our PPI contributors and S. C.‐S. via Zoom. We made amendments to the language in the introduction section as well as amendments to the questions in our topic guide to account for language, translation and communication aspects of data collection. In addition, we were advised by our PPI collaborators to offer participants the option of taking part in a non‐English language‐speaking focus group, in a familiar and safe, community setting. The groups would be facilitated by a PPI contributor who speaks the relevant language to allow for the best opportunity to collect the data in the most culturally sensitive way. Both T. M. and N. A. had prior experience working with community groups and facilitating focus groups. Of note, TM had experience in transcribing, interpreting and translating SA languages into English. PPI collaborators were reimbursed for their contributions per hour in line with INVOLVE rates.^32^

### 2.3 Setting

Focus groups were conducted, face‐to‐face, in SA community settings in areas populated with people from ethnic minority backgrounds to allow for maximum variation amongst practice size, location and deprivation within Greater Manchester (GM) in the United Kingdom. These took place at the SA community centres where the PPI contributors already had links with the organisation. A mix of men and women‐only SA organisations participated to give a mix of characteristics, such as sex and age. Individual interviews were all conducted remotely via Zoom and Microsoft Teams or telephone by SCS or NS.

### 2.4 Sampling

Individuals who self‐identified as being within the SA community (Indian, Pakistani, Bangladeshi or Sri Lankan background), who were over the age of 18 years of age, who had one or more appointments with their local General Practice (GP or nurse) over the previous 12 months or more during the COVID‐19 pandemic, and who consented to participate in the research were eligible. We sought to recruit 8–10 participants to each single‐sex focus group.^33^

### 2.5 Recruitment: PPI/community led

Our PPI and previous evidence^34^ directed our approach ensuring that rapport, trust, and respect for religious/cultural knowledge were central to an inclusive and culturally sensitive approach. PPI members led all focus groups in the participants' preferred language. Multiple approaches to recruitment were adopted to ensure maximum variability at the participant level and consequently, participants utilising differing access/consultation systems within their practices were included. Recruitment to the study took place between March and December 2022. We prioritised recruitment to non‐English speaking community focus groups initially. T. M., N. A. and H. H. recruited all the participants for the focus groups by approaching 6 SA community organisations across GM. This involved scheduling either an in‐person meeting, or a telephone call followed by an email, depending on the preferred method of contact the PPI contributor had in place with the SA organisation. The patient‐facing materials were either printed and taken to the in‐person meeting to circulate, or attached to an email for potential participants to contact the researchers directly.

Interview participants were recruited from GM community settings, Twitter and existing and public involvement groups/leads by H. H., T. M., N. A. and N. S. Interested participants were given a consent to  contact form requesting personal contact details and spoken/written language preferences. Potential participants were emailed or given in person the full Participant Information Sheet, consent form and consent to contact form, depending on preference. People indicated their preferred language for spoken and written language so we could allocate people to either a non‐English or English language focus group or English language interview.

### 2.6 Recruitment: Researcher led

N. S. and S. C.‐S. also recruited individuals from practices using a digital access and consultation system called PATCHS.^35^ Recruitment was via a Nationwide database of patients who had consented to be contacted by University of Manchester (UoM) researchers by email about further research. This enabled us to sample people who had experience using the PATCHS system, who were of SA background and interested in taking part in further research. Initially, N. S. and S. C.‐S. emailed the advert, participant information sheet and consent form to 50 patients who were recorded as having SA ethnicity. Subsequently, a mass mail merge was conducted to 3264 patients. Those who agreed to participate consented via email. Nationwide recruitment facilitated a wider range of geographical/local factors to potentially be included in the sample versus recruitment PPI‐led recruitment within GM.

### 2.7 Data collection: In‐person focus groups

Homogenous groups with a common language (one in Urdu, Bangla and English respectively) and the sex of participants were selected to facilitate ease of communication and a synergy of ideas from the participants in the group.^33^ In‐person groups were also preferred due to the value that people from SA place on nonverbal aspects of communication and that nonverbal communication (NVC) styles may vary across cultures.^36^ Groups within each SA community organisation, therefore, examined participants' experiences of access, triage and care in general practice. Using the same multilingual PPI collaborators, T. M. and N. A., to recruit and co‐facilitate the focus groups helped participants to develop trust and rapport, with the aim of making them feel more comfortable with the research. Participants were welcomed and introduced to PPI collaborators and researchers in attendance (N. S., S. C.‐S. and Y. M.). Participants were then briefed about the study, how the focus groups would be conducted and were reassured about their privacy. Verbal and written informed consent to take part in the study was taken by the multilingual researcher, Y. M. in the case of non‐English speaking groups and taken by N. S. for the English‐speaking group. Demographics were collected at the end of the group/individual interview.

Our PPI collaborator (T. M.) facilitated the discussions with researchers using the topic guide to steer the discussion but also to explore interesting lines of inquiry and enable free expression of views. In line with ethics, our multilingual research (Y. M.) also attended the non‐English speaking groups to ensure consent procedures were formally observed and followed a discussion in real time allowing for lines of communication across the collaborative team. The topic guide (developed from the literature and PPI involvement in the co‐development and piloting: see Section 2.2 for detail) progressed through pre‐ and ‘post‐COVID’ (hereafter we use the term ‘post‐COVID’ to indicate the period since May 2021 when the UK government lifted mandated remote access and consultation restrictions and outlined that face‐to‐face consultations ought to be routinely offered by way of a return to ‘normality’) experiences of access and care, to understand perceptions of the changes, experiences with new modes of access, care and their perceived outcomes including the quality and safety of care (File S1). Comments, thoughts and feelings of individuals were explored rather than seeking to gain only a consensus view.^33^ Y. M. and N. A. took notes on the content of the discussions. The content of the dialogue was transcribed verbatim by T. M. which aided the representation of the meaning of interpretation of what was discussed within the groups. The translated transcript was back‐checked by multilingual UoM researchers and a UoM medical student to make sure the transcript was accurate and reflected the thoughts, views and opinions appropriately.

### 2.8 Remote interviews

Due to nationwide recruitment and resource constraints, interviews were conducted in English and remotely (digitally or via telephone) by N. S. and S. C.‐S. according to the preference of the individual. Recordings were transcribed via a professional transcribing company. The initial topic guide for interviews largely mirrored the major areas for that of the focus groups but was adapted in line with the emergent findings from the focus groups to follow up on those general issues identified within the groups and allowed for more in‐depth discussion of those issues.

### 2.9 Analysis

Data collection and analysis were conducted iteratively. Interview, focus group transcripts, and observational notes were pseudonymised on receipt. Thematic analysis^37^ was conducted to identify, analyse and report patterns in the qualitative data. This was an iterative process whereby initial themes within the focus groups guided the exploration of individual interviews enriching the data. Transcripts were independently read by two researchers (S. C.‐S., N. S.) who, once familiar with the breadth and depth of content, undertook open coding in NVIVO. Ideas for themes were generated from the initial coding and then grouped under broader categories through discussion. PPI collaborators were invited to review the themes and provide feedback.

## 3 FINDINGS

Thirty‐seven participants were recruited, and data collected from three focus groups recruited across three SA communities within GM and 11 semistructured interviews (*n* = 2: recruited from two SA communities via Twitter/PPI Leads and *n* = 9 from the PATCHS nationwide database) were conducted between June and November 2022 (see Table 1). Focus groups were on average 64 min in length and interviews were on average 40 min (range 26–53 min).

**Table 1:** Avenues of recruitment for focus group and interview participants and composition.

Recruitment avenue/focus group (FG) ID	Number of in‐person focus group participants, SA ethnic group, sex, preferred spoken language	Number of remote interview participants in the English language
SA organisation for men, FG1	8, Bangladeshi, men, Bangali speaking	0
SA organisation for women, FG2	8, Urdu women, Urdu speaking	0
SA organisation for women and men, FG3	10, Bangladeshi and Urdu women, English Language	0
Other community SA organisations via Twitter/PPI leads	0	2
PATCHS nationwide database with SA mail out	0	9
Number per interview type	26	11
Total number of participants	37	

Abbreviation: PPI, patient and public involvement; SA, South Asian.

The demographic characteristics of each participant are illustrated in Table 2. Two non‐English speaking focus groups were conducted (Bangla [*n* = 8 male participants] and Urdu [*n* = 8 female] languages) and one was in English (*n* = 10 female participants). The majority of interview participants in the study were of Pakistani (*n* = 5) or Indian (*n* = 5) ethnicity. The sample overall was 59% female (22/37), median ages 45–54 years (range 25–84 years) and 56% were unemployed, with 33% self‐reporting as a carer. Regarding health during COVID‐19 restrictions, 42% of people self‐reported living with a long‐term condition, with 44% choosing to shield and avoiding making a GP appointment during this time (22% reported finding this too difficult). Of note, 9/37 participants would prefer any health information in a language other than English, and 4 indicated a preference for English or another language, in this case, Urdu. The vast majority of participants were digitally enabled as they indicated smartphone ownership and over half, had a PC at home with a Wi‐Fi connection.

**Table 2:** Participant demographics of focus group and interview participants.

Participant ID	Focus group (FG) ID/interview ID	Age	First half of postcode	Gender (male [M] /female [F])	SA ethnic group	Employment	Carer (hours per week)	Living with a long‐term condition (yes [Y]/no [N])	Shielded during COVID‐19 (Y/N)	Avoided making GP appointment during COVID‐19 (Y/N)	Language preference for written health information/spoken health information	Own a PC at home with Wi‐Fi (Y/N)	Own or access to a mobile/own or access to a smartphone Y/N)	Family/friend looked for health info online for you during COVID‐19 (Y/N)
506	FG1	45–54	OL12	M	Bangladeshi	PT paid	Y (20–34)	Y	N	N	Bangali/Bangali	N	Y/Y	N
507	FG1	45–54	OL16	M	Bangladeshi	PT paid	N	N	Y	Not needed one	Bangali/Bangali	N	Y/Y	N
508	FG1	65–74	OL16	M	Bangladeshi	Retired	N	Y	N	Y, too difficult	Bangla/Bangla	N	Y/Y	N
509	FG1	65–74	OL16	M	Bangladeshi	PT paid	N	N	Y	Not needed one	Bengali/Bengali	N	Y/Y	N
510	FG1	35–44	OL16	M	Bangladeshi	Unemployed	N	N	N	N	English/English	N	Y/Y	N
511	FG1	75–84	OL11	M	Bangladeshi	Retired	Y (1–9)	N	Y	N	Bengali/Bengali	N	Y/Y	Y
512	FG1	65–74	OL16	M	Bangladeshi	FT paid	N	N	Y	Y, didn't have time	English/English	Y	Y/Y	Y
513	FG1	35–44	OL11	M	Bangladeshi	PT paid	N	N	N	N	English/English	Y	Y/Y	Y
501	FG2	35–44	M32	F	Pakistani	Looking after family	Y (20–34)	Prefer not to say	N	N	Urdu/Urdu	N	Y/No answer	Y
*Note*: FG 1–2 composition/language: FG1 = Bangladeshi men; FG2 = Urdu women														
503	FG2	45–54	–	F	Pakistani	Doing something else	N	Prefer not to say	N	Y, too difficult	English/English	N	Y/Y	N
504	FG2	45–54	–	F	Pakistani	Doing something else	N	Y	Y	N	Urdu/Urdu	Y	Y/Y	N
505	FG2	35–44	–	F	Bangali	–	–	–	–	–	English/English	–	–/–	–
506	FG2	55–64	BL9	F	White and Asian, Pakistani	–	–	Y	N	Y, too difficult	Urdu/Urdu	Y	Y/Y	Y
507	FG2	25–34	–	F	White and Asian, Pakistani	Unemployed	N	Y	Y	Y, catching covid, too difficult	English/English	N	Y/Y	N
508	FG2	35–44	M14	F	Pakistani	Unemployed	N	Y	Y	Y, catching covid,	English/English	N	Y/Y	Y
521	FG2	55–64	OL11	F	Pakistani	PT paid	–	Y	Y/Y	Y, for another reason	English, Bangla, Urdu/English, Bangla, Urdu/	Y	Y/Y	N
*Note*: FG2 composition/language: Urdu, women; missing data = –														
522	FG2	75–84	OL11	F	Pakistani	Looking after family	N	Don't know	N	Y, too difficult	Urdu/Urdu	–	Y/Y	N
523	FG3	35–44	OL12	F	Pakistani	PT paid	N	N	N	Y, worried re burden on nhs	English/Urdu	Y	Y/Y	N
524	FG3	45–54	OL12	F	Pakistani	PT paid	Y (10–19)	Y	N	N, ‘booked telephone appointment when could not access GP surgery’	English/English	Y	Y/Y	N
525	FG3	45–54	OL16	F	Pakistani	Looking after family	–	Y	Y	Haven't needed one	Urdu/Urdu	Y	Y/Y	Y
526	FG3	45–54	OL11	F	White and Asian, Pakistani	Looking after family	N	Y	N	N	English/English	Y	Y/N	N
527	FG3	35–44	OL11	F	Pakistani	PT, paid	Y (1–9)	N	N	Y, for another reason	English, Urdu/English, Urdu	Y	Y/Y	Y
528	FG3	55–64	OL16	F	Pakistani ‘British’	Unemployed	Y (50+)	Y	Y	Y, too difficult	English/English	–	Don't know/Y	Y
529	FG3	55–64	OL11	F	White and Asian, Pakistani	PT paid	N	N/don't know	N	N	English/English	Y	Y/Y	N
*Note*: FG2–3 composition: language, gender: FG2 = Urdu, women; FG3 = Pakistani, women; missing data = –														
530	FG3	45–54	OL11	F	Mixed, Pakistani	Looking after family	Y (50+)	Y	Y, (some‐one in my home)	Y, I didn't have time	English/English	N	Y/Y	N
531	IV^a^	45–54	OL8	F	Pakistani	Looking after family	Y (35–49)	N	N	N	English/English	Y	Y/Y	N
532	IV^a^	45–54	OL8	F	Pakistani	FT paid	Y (10–19)	N	Y	Y	English/English	Y	Y/Y	N
533	IV^b^	25–34	NW4	M	Indian	FT paid	N	N	Y, shielded	N	English/English	Y	Y/Y	Y
534	IV^b^	35–44	NW4	M	Asian British	Permanently sick or disabled	N	Y	Y shielded	N	English/English	N	Y/Y	Y
535	IV^b^	45–54	NW4	F	Indian	PT paid	Y (1–9)	Y	N	N	English/English	Y	Y/Y	Y
356	IV^b^	55–64	HA4	M	Indian	FY paid	N	N	N	Y, worried about the burden on NHS	English/English	Y	Y/Y	N
*Note*: FG3 composition: language, gender = Pakistani, English language group, women														
357	IV^b^	65– 74	NW9	M	Indian	Retired	Y (20–34)	Y	N	N	English/English	Y	Y/Y	Y
358	IV^b^	45– 54	L18	F	Pakistani	Looking after family	N	N	N	N	English/English	Y	Y/Y	N
359	IV^b^	35– 44	UB7	M	Pakistani	FT paid	N	N	Y	Y, too difficult	English/English	N	Y/Y	N
360	IV^b^	25–34	M6	M	Pakistani	Unemployed	Y (10–19)	N	Y	Y, too difficult	English/English	Y	Y/Y	N
361	IV^b^	35– 44	SN5	M	Indian	FT paid	N	N	N	N	English/English	Y	Y/Y	N
*Note*: FG3 Focus group composition: language, gender = Pakistani, English language group, women														

Abbreviations: FT, full time; IV, Interview; PT, part time.

^a^ Community participant.

^b^ PATCHS participant.

Three major themes were identified. Illustrative data are given and identified by a participant identification number, which is provided along with contextual information on sex, participant group (community/PATCHS), age range in years and interview type (focus group/interview).

### 3.1 Reduced access

This theme was split between general access issues that were not ethnicity and/or language‐related and those that were.

#### 3.1.1 General access issues: Telephone and technical barriers

Patients compared and contrasted their pre‐ and postpandemic restriction experiences of accessing primary care and all described a perceived reduction in accessibility owing to the pandemic ‘post‐COVID’ restrictions. Participants depicted pre‐COVID, 2020 access to primary care services as relatively simple, responsive and easy to navigate (vs. current means) with access via one of three ways, a request through telephone contact, in‐person (walk‐in request) or less often, via a simple online system or digital app:

with our GP practice you have to ring up between eight and nine if you want an appointment and if you want to prebook an appointment, then you can phone up and request a prebook appointment which will be in a couple of weeks’ time. So that was generally fine, and appointments were fairly good, available. We didn't really have a problem getting an appointment and the doctor would see you face‐to‐face. So that was a positive experience before the COVID came in. (ID532, female community participant, 45–54 years, English language interview)

Since mandated COVID restrictions were lifted however, all participants described worsening accessibility and overall, less responsive care. Telephone access was the most prevalent and preferred access mode for the majority of participants but is also now seen as less accessible than in the pre‐COVID era due to the perception of decreased appointment availability. All participants understood how to navigate the system (e.g. call as the surgery opens at 8:00 AM) to try and make an appointment, but went on to describe areas of difficulty with the process; (1) ‘getting in the queue’, (2) ‘staying in the queue’ and (3) extensive queueing times, all leading to difficulties for those with childcare or work responsibilities:

I return home late in the night from my job and can't wake up so early to call at 8am. (ID506, male participant, 45–54 years, Bangali speaking focus group 1)

I was working on the computer, my phone was on speaker [whilst queuing], but luckily I didn't have any work meeting at that time, but if that is the case, then I probably have to cut the phone down and finish my meeting off. (ID361, male PATCHS participant, 35–44 years, English language interview)

However, queuing did not necessarily result in the patient receiving an appointment due to a lack of appointment availability once they reached the front of the queue and therefore the process would need to begin again the following morning creating the perception of increased patient efforts to access care often with no successful outcome:

It's a waste time for me wake up in the morning, hold on to the line for an hour, when I eventually get through the receptionist will say oh, we don't we don't have any appointments. (ID506, male participant, 45–54 years, Bangali speaking focus group 1)

If appointments were available, many described a new ‘post‐COVID’ step and in many cases a new step in the access process, telephone triage. Telephone triage was new for the vast majority of participants and in most cases, this was predominantly conducted by practice receptionists. The triage process was however not well understood or received by some participants who lamented a lack of communication from practices to their patients as well:

Now when I ring the same … exactly the same problem but now when you ring they actually ask you what the … you know, what it's for, yeah, You know, she never used to ask you before for what the reason is, or whatever, or, you know? (ID532, female community participant, 45–54 years, English language interview)

Should triage (digital or telephone) result in a consultation being required, patients then described awaiting a call back from a clinician. Some participants were able to accommodate the callbacks more easily than others due to the variation in personal and social circumstances, with those in work expressing the greatest issues. All however expressed anxieties around missing the call and along with it their opportunity to receive care:

When I have called from home, they say they will call back, when they call, they only ring once or twice then they disconnect. I don't even go to the bathroom fearing I'll miss the call. (ID506, male participant, 45–54 years, Bangali speaking focus group 1)

Digital access systems rolled out during the pandemic were seen as having both positive and negative outcomes for access. The vast majority of participants were digitally ‘enabled’ (see Table 2) but a minority of participants were unaware of the possibility of the option of digital access (e.g., for booking/requesting appointments). Of those participants who had experience with digital access, most found the system easy to navigate and more convenient for making appointments than telephone access due to their flexibility and asynchronous nature allowing for easier and more convenient appointment‐making and communication:

I used to call to the GP sometimes it takes five minutes, sometimes 15 minutes, sometimes half an hour to just pick up the phone. That's why I think I prefer, like, online system to book online rather than over the phone. (ID533, male PATCHS participant, 25–34 years, English language interview)

Those participants that lacked awareness or were aware but lacked the confidence to utilise digital systems attributed this to digital literacy issues either for themselves or others that they cared for (primarily older relatives). The need for support from others for digital access was highlighted. Support was required particularly for older participants and those with elderly relatives who were seen as most at risk of exclusion from this access route:

I have a laptop and I have ways of using, I know how to use. I can't say for people who are not that comfortable with using technology and all that. But myself, my family, the younger, my children, they're fine. But for my mother‐in‐law for example she wouldn't be able to do that independently; she would definitely need somebody with her. (ID535, female PATCHS participant, 45–54 years, English language interview)

#### 3.1.2 Specific access issues: Communication barriers and staff attitudes

Some access issues were specifically related to participant language and/or ethnicity which created a perception of additional barriers to access for some participants. First, a minority of participants from the non‐English speaking groups spoke of issues that they related specifically to their limited proficiency in the English Language (LEP). Other participants however also referred to these issues for family members with LEP, often older relatives. Communication difficulties stemming from LEP ran throughout the experiences they recounted and ranged from their initial contact attempts via telephone access through to the consultations themselves.

First, language issues meant that telephone access was seen as the only feasible access route. Digital options were seen as inaccessible for those with LEP unless someone else could make the appointment request for you:

And the apps don't even accommodate, they definitely don't accommodate any kind of translation, or having a translation through the app. (Patchs does have a translation function, however as highlighted earlier, it may be that this was not turned on in this practice.) (ID360, male PATCHS participant, 25–34 years, English Language interview).

Using this mode of access however was also difficult for those with LEP, particularly when communicating with receptionists (face‐to‐face or via telephone).

Even when I have got through to them, I have struggled. I don't understand Urdu and not very fluent in English, I speak with a mix of English and Bangla. But they [receptionists] don't understand me, they don't wait for me to finish, they put the phone down. (ID506, male participant, 45–54 years, Bangali speaking focus group 1).

Some participants recounted particular difficulties in access beyond LEP however, attributing issues not only to language difficulties but also to negative experiences of receptionists' staff attitudes towards themselves and/or others, with a very small minority alluding to perceived differences in their treatment due to their LEP or ethnicity:

it depends on the people, I wouldn't say they are good or bad… if you speak English and they understand, they will actually, I mean, they will treat you well. But if you don't, and sometimes… I haven't actually noticed it but, say, suppose there was a very old lady, she was not able to talk and she was Indian as well. So, I was there, she was trying to explain something to the, you know, the people who were actually there for admin and she was not very nice to her. (ID533, male PATCHS participant, 25–35 years, English language interview).

Translator availability (both formal and/or informal via a relative) was seen an additional access barrier to care once they received a consultation:

now if you, if you get to see a doctor and she needs a translator then it should be arranged as well. So that becomes kind of a barrier as well. (ID512, male participant, 65–74 years, Bangla speaking focus group 1)

Well, yeah, when she's trying to communicate [mother], sometimes it's hard to get a doctor that can help or get a translator she's told me, so then one of us will have to always go to appointment, which is a bit hard when we're working. (ID359, male PATCHS participant, 35–44 years, English language interview)

Where staff were ethnically concordant and/or linguistically competent in terms of the participants preferred language, these issues were largely removed. However, a very small minority of participants felt that ethnicity was a factor in the way they or others were dealt with, particularly by reception staff:

We had a Bengali madam, can't remember her name, she was quite nice, even if she didn't have time she would talk politely. She told me to call between a certain time and she would try to get me an appointment. But in my area, most of the staff are our Pakistani sisters. Whenever I got to them, they say, call us, don't come here whenever you want. I told them you don't receive calls what can I do? They say, call that all, they don't talk to us after that. (ID506, male participant, 45‐54 years, Bangla speaking focus group 1)

I've found myself, if I actually go to see a GP and they're Indian they will be a bit rude. If they are from other…you know, maybe anywhere or maybe British or maybe other ethnicity, they will be quite nicer than that. Yeah, I don't know why, it's very, very, weird even though, you know, people are supposed to be very nice to each other regardless what ethnicity you are. But I've found a bit rude when you see a GP or when you see an admin who is working or they're Indian or Asian. (ID533, male PATCHS participant, 25–34 years, English language‐interview)

### 3.2 Reduced patient choice

All participants described a reduction in choice ‘post‐COVID’ across different areas of access and care. First, many participants expressed a preference for face‐to‐face consultations but also stated that they did not receive this, with telephone consultations being described as the primary means of accessing and receiving medical care. Face‐to‐face options were perceived as largely unavailable or rationed and difficult to access even when they expressed a preference for receiving this during triage. Remote means of accessing care were acceptable and even preferable for some participants however, but others felt they should be apportioned according to the perceived need as defined by the patient:

So, let me explain. If I've got a cold and things, which has been going for a week or so, it may be sufficient to have a video or audio call and go through it. But if I've got a chronic condition that, you know, and I needed reassurance and things, I think then the option for a video as well as a face‐to‐face should be given. (ID357, male PATCHS participant, 65–74 years, English language interview)

Many participants expressed frustrations and concerns about a perceived lack of their preferred mode of face‐to‐face care which they valued for multiple reasons with most placing a high value on interpersonal factors associated with face‐to‐face care that went beyond clinical competence and was equated with better care:

I don't want to be talking to a screen. It is a big deal, you know. There is some comfort in that face‐to‐face kind of chat because your doctor was also your friend when it came to your health. (ID521, female participant, 55–64 years, Pakistani/English speaking focus group 3)

if we meet face to face it's the body language you can read, is very important for patients and trust and the confidence and the relationship with the doctor and the patient. (ID358, female PATCHS participant, 45–54 years, English language interview)

Second, there was a perceived lack of choice of who they saw, from the type of practitioner (e.g., nurse or GP) to a specific preferred doctor leading to perceptions of reduced continuity:

it's very hard to see a GP, and I've noticed that now they try to just give it to a nurse, paediatric nurse, not really seeing a GP anymore, or if they can, just try to do it over the phone, they're still not really giving that many face‐to‐face appointments, very rarely, or you have to be quite demanding to see a GP I believe. (ID359, male PATCHS participant, 35–44 years, English language interview)

even though I have been requesting to meet him as he knows my case history. Every time I request for him, they say they are not available, you must take whoever is available. (ID521, female participant, 55–64 years, Pakistani/English speaking focus group 3)

### 3.3 Quality and safety

#### 3.3.1 Reduced responsiveness and trust

The perceived reduction in accessibility and use of remote means of access and care were often associated with a service that was seen as less responsive to patient need, leading to delays in various areas of care. This in combination with the perceived reduction in face‐to‐face care also created concerns amongst some participants for the quality and safety of care they had received:

So as a carer I was sending pictures of my mother who had a swollen foot and it was really bad and they kept on diagnosing her but they weren't giving us the real diagnosis. So it was only when we got in touch with the walk‐in, the seven‐day access, they actually did a phone consultation and said, right, okay, you have to get her into hospital, where our GP was not, kind of, responding positively in a way. He wasn't diagnosing of what's going on and he wasn't getting her seen. (ID532, male community participant, 45–54 years, English language interview)

Remote access to care was often perceived by many participants to be less ‘good care' due to the removal of a large element of psycho‐social safety that face‐to‐face consultations were perceived to provide. Participants valued the reassurance and ability to trust the outcome of the consultation due to ‘being seen’:

I mean, you're describing it verbally and sometimes there are certain things that are much better in your visual, they need to check something, no matter what, video would be slightly better but I still think sometimes it's better to see personally. (ID361, male PATCHS participant, 35–44 years, English language interview)

if you go and see in person, I mean, you feel more secure, first, and then you feel like you're in good hands. (ID533, male PATCHS participant, 25–34 years, English language interview)

Being seen face‐to‐face was seen as particularly important for those that were considered to be vulnerable (e.g., elderly or children) or require complex care:

Recently I had to make an appointment for my mother‐in‐law to go and see them, and in her case, she was offered a face‐to‐face appointment, and I could go with her. But for myself no. (ID535, female PATCHS participant, 45–54 years, English language interview)

#### 3.3.2 Reduced safety due to LEP: Remote losses and consequences

For those with LEP or supporting those with LEP, there were concerns for the quality and safety of remote care which centred around communication and their ability to interact with staff when attempting to access and receive care. The first communication issue related to translation. Here concerns were associated with 1) the perceived difficulties of interpretation via formal translators and 2) the limitations in some clinical encounters by professionals on the use of relatives (their preferred method of communication). The use of formal interpreters was seen as less preferable to a person within their personal social network (usually a close relative) who was perceived as able to more accurately convey their health concerns and/or prompt issues of relevance:

Yeah, they want it to be an interpreter but then you find that the interpreter might not be able to exactly describe what the person is trying to say. (ID534, male PATCHS participant, 35–44 years, English language interview)

I like that [face‐to‐face consultations] because you build a rapport with the doctor and things and then also it meant that the wife could come and then there's two ears listening to it. And if you forget to ask something she would get to ask some questions or the other way round. (ID357, male PATCHS participant, 65–74 years, English language interview)

Second, remote care removed an important aspect of communication, namely nonverbal aspects which were seen as particularly important for those with LEP. Links were also made to the need for cultural understandings which go beyond language and were important culturally:

A bit example is with my wife, I mean if you actually asked her maybe with two lines she would say what exactly happened with her, so you wouldn't actually understand. So if you saw her in person then you would actually have a big difference because you will understand, oh this person is this and that. You understand? (ID533, male PATCHS participant, 25–34 years, English language interview)

they should have at least one to two doctors who understand the cultural background of the, it doesn't matter how much we educate them about our culture. At the end of the day they do not know. Only a person who comes from the same background will understand. (ID358, female PATCHS participant, 45–54 years, English language interview)

Quality and safety concerns associated with a perceived lack of face‐to‐face care (e.g., ability to be examined or ‘seen’) led to some participants occasionally and in a minority of cases, routinely, bypassing general practice and seeking care from alternate sources such as Accident and Emergency (A&E):

A couple of things happened but then I went to A&E, because I knew that my GP's not going to be able to help me this quickly, so I chose to wait in A&E for several hours because I wanted to be seen by someone, they checked me and did some blood tests and everything. (ID535, female PATCHS participant, 45–54 years, English language interview)

Most participants were however largely satisfied with the outcome of the care they received, once they had navigated contact through to the clinical team and during the consultations themselves:

Whatever problem I had, I think I had more with the booking system but once I get through to someone, the nurse or somebody, at least they see you or they try to solve the issue or whatever. (ID361, male PATCHS participant, 35–44 years, English language interview).

For some who were able to access and use digital systems, these were preferable for communication, as they were able to construct the nature of their problem without the pressures of being in a ‘live’ consultation as well as written communication providing an audit trail of information:

it stores everything, and yeah, like in time order, date order…[so] there's no misunderstandings. In the old days you might have said, I said this, that, and the other, and then the other side is saying, well you never said this, that, and the other. Now it's clearly written down, you've had your opportunity to write down exactly what the problem was, and then they're working off what you've written. (ID360, male PATCHS participant, 25–34 years, English language interview)

## 4 DISCUSSION

Our findings suggest SA patients are experiencing multiple difficulties in accessing and receiving primary care services since COVID‐19 and the continued use of newer models of access to, and delivery of care. Participants experienced similar concerns regarding a lack of timely and preferred care modes ‘post‐COVID’, as reported by other patient groups. However, we identified issues that are perceived to exacerbate and create new care inequities for a subset of SA patients.

Pre‐COVID evidence suggested a trend of reduced patient satisfaction both overall in terms of experience and in accessing an appointment itself.^12^, ^13^ Dissatisfaction and consultation rates were highest and lowest respectively amongst Asian patients^38^ and our findings show SA participants perceptions are of a less accessible and responsive service versus pre‐COVID. As found elsewhere, remote access and triage were valued by some participants for its convenience,^39^ but were also seen as limited in multiple ways, particularly for older SA adults and those with LEP. Participants felt that the use of remote services ought to be limited to certain (minor) care scenarios or patients/groups.

The continued use of remote means of accessing and receiving care since COVID‐19 has seemingly led to the creation or exacerbation of access and care issues creating a particularly poorer experience of care delivery ‘post‐COVID’ for a minority of SA participants, particularly those with LEP. For these participants, there is seemingly a situation of increased inequity in access and care processes. This assertion is supported by studies that suggest patients with an Asian background were less likely to receive face‐to‐face care during the pandemic^40^ and a disproportionate reduction in face‐to‐face consultations may negatively impact the perceptions of the quality of care received by individuals of Asian ethnicity as reported by some participants in this study. Furthermore, those with LEP may be able to *speak* English (to varying extents) but they might not be able to read or write it therefore limiting accessibility and possible routes to care that rely on digital access means. This study suggests that for some SA patients, an intersection of issues which include language and culture barriers,^14^ and preferences for face‐to‐face care (particularly important for those with LEP), combined with digital and health literacy issues, has created a perception of exacerbated inequalities for access under remote triage models, with those with LEP reporting a poorer ability to navigate newer access and care models (e.g., telephone/digital triage and remote consultations). A lack of accurate and timely interpretation also created uncertainty and concerns for the quality and safety of care and trust in the processes and outcomes. Remote consultations amplify these concerns, removing important and valued aspects of NVC and psychosocial safety.^41^

Accounts of patient disengagement with, and from primary care due to access issues may have severe consequences for individuals as well as the wider system both in social and economic terms. Such conclusions may be drawn from evidence gathered from other groups who bypass primary care albeit for differing reasons.^42^ Furthermore, ethnic minority groups experience a higher burden of some conditions that are potentially preventable and perceptions of reduced access and poorer experiences of care may lead to increases in preventable emergency hospital admissions for this group of patients.^43^

Good communication is an essential skill and core feature of good quality general practice.^44^, ^45^ Our findings in a UK sample also emphasise the importance of communication skills for patients' use and experience of accessing and using new models of care resonating with International studies^10^, ^11^, ^12^. Further, they underpin the importance of psychosocial aspects of safety, such as continuity of care, especially personal continuity, to overcome some of the barriers related to trust commonly experienced by minority ethnic groups.^9^

Practices need to be cognisant of the importance of SA patient communication and support needs to ensure patients with specific cultural beliefs are equitably able to access their services.^14^ Largescale changes to service access, need to be accompanied by better patient education,^46^ but it is understandable that this was perhaps less of a priority during the pandemic in this case. Furthermore, staff, particularly those who have first‐patient contact (e.g., reception staff), may require training around LEP and cultural competency to ensure that services are equitable and to understand reasons for disengagement (such as preferring to seek informal care vs. formal care).^17^ In particular, NVC has been shown to be of particular value to those from SA communities^36^ and should be taken into account when designing and evaluating models for accessing and using health care as well as staff training to ensure cultural competency. The role of ethnic and/or language concordance or discordance within the access, triage and consultation communication and outcomes process also required further exploration. With some studies suggesting improved patient experience when practices have the ability to offer a language concordant with SA patients’ ethnicity.^47^ Furthermore, if online consultation tools do provide translation capabilities that are not being activated by practices, this creates further and unnecessary barriers to access. Further research is required to understand the balance of risk between the potential for translation errors via digital tools and/or the access barriers created by their use without such features. Finally, whilst research has focused on digital exclusion in the forms of digital literacy and poverty, this has potentially excluded more basic modes of exclusion and barriers to access under new digital forms of access and care, such as language concordance/fluency and literacy.

### 4.1 Strengths and limitations

Our study findings are supported by a relatively large sample, comprised of multiple SA ethnicities, languages (English and non‐English), a range of ages and digital experience/enablement. We also had strong PPI involvement from the outset and throughout the project, allowing access to people who may not normally be aware of research opportunities. For this reason, we were able to recruit successfully with most success coming from our PPI contributors. The mixture of face‐to‐face and remote methods allowed for a wide range of participants as well as a wide geographical range to be covered, but the email mass mailouts via a nationwide database proved a poor form of recruitment method (with 27% successfully recruited) and reinforces the need for culturally appropriate methods of recruitment^34^ and good PPI embedded throughout the study as in the current study.

We acknowledge however that Pakistani and Bangladeshi communities had the poorest outcomes before COVID‐19^48^ and the ethnic minorities overall suffered a disproportionate impact of COVID‐19 and therefore the negative views within this study may reflect that experience but also emphasises the importance of the need for this research study and its findings. It is also likely that the relative dissatisfaction expressed here may also be due to deprivation. People from minority ethnic groups are, on average, much more likely to be in income poverty than white British people. Within ethnic minorities, those from Bangladeshi or Pakistani backgrounds have the highest rates of income poverty^24^ and made up a substantial proportion of our study sample. Furthermore, areas of GM are relatively deprived. People on lower incomes may not have equitable access to digital services and or phone/internet credit to make appointments or spend long periods of time queuing on the phone. In other words, a lack of digital connectivity can now potentially lead to impoverished health and increased inequality via reduced access to care. The majority of people in our sample were however digitally enabled via smartphones. We were however unable to assess their level of connectivity beyond this. Those in the lowest socioeconomic bands and those with LEP are also more likely to have poorer health literacy and literacy levels, and 5 million people in the UK lack literacy and numerical literacy skills.^49^ From a service provision perspective, it is difficult to disentangle the effect of deprivation, with poorer areas having less provision^50^ making de facto access harder. It is clear, however, that some issues raised in our study here do not pertain to deprivation, but to LEP and cultural differences, and potentially religious views. Of note, we did not specifically ask participants about their religious views/beliefs at the interview or in the demographic questionnaire. We were unable to ascertain the deprivation levels of individual participants which may affect their perceptions as those from more deprived areas have poorer experiences of their GP practices however pre‐COVID SA patients describe poorer experiences relative to white and black patients.^38^ This trend may have continued and even exacerbated since COVID‐19, only further work will ascertain whether this is the case. Finally, we were unable to ascertain the range of digital systems used with only one known for certainty; however, the focus was on general issues of accessing and receiving care via such systems and not system‐specific issues.

## 5 CONCLUSION

Rapid implementation and a more permanent post‐COVID move to a remote‐led triage system have led to SA patients negatively describing their experiences of accessing and using primary care services. Whilst some participants viewed remote access positively, this was limited to a small subset of participants and/or for certain scenarios. Face‐to‐face models of caregiving remain the preferred mode of consultation, particularly for those with LEP. Hybrid models of access offer patients the greatest choice and are likely to meet the varying needs of the SA patient population going forwards. The digital‐first approach to primary care may be achievable as a service ideal, but its limitations need to be recognised and accounted for to ensure that primary care can be an equitable service, both now and in the future. Digital healthcare content needs to be designed for an ethnically diverse audience, otherwise it has the potential to exclude them by not taking their needs into account.

## AUTHOR CONTRIBUTIONS

**Nicola Small**: Writing—original draft; writing—review and editing; formal analysis; project administration; resources; data curation; methodology; software; supervision; investigation. **Yumna Masood**: writing—review and editing; methodology; data curation; supervision; project administration; formal analysis; resources. **Fiona Stevenson**: Conceptualisation; funding acquisition; writing—original draft; writing—review and editing; methodology; formal analysis; investigation. **Benjamin C. Brown**: Conceptualization; investigation; funding acquisition; writing—original draft; writing—review and editing; formal analysis; methodology. **Caroline Sanders**: Conceptualisation; investigation; funding acquisition; writing—original draft; writing—review and editing; methodology; formal analysis. **Brian McMillan**: Conceptualisation; investigation; funding acquisition; writing—original draft; writing—review and editing; methodology; formal analysis. **Helen Atherton**: Conceptualisation; investigation; funding acquisition; writing—original draft; writing—review and editing; methodology; formal analysis. **Tandrima Mazumdar**: data curation; resources; project administration; formal analysis; writing—review and editing. **Nigat Ara**: Data curation; resources; project administration; formal analysis; writing—review and editing. **Humera Haqqani**: Data curation; resources; project administration; formal analysis; writing—review and editing. **Sudeh Cheraghi‐Sohi**: Conceptualisation; investigation; funding acquisition; writing—original draft; methodology; validation; visualisation; writing—review and editing; formal analysis; software; project administration; resources; supervision; data curation.

## CONFLICT OF INTEREST STATEMENT DISCLOSURE

Benjamin C. Brown is a part‐time employee of Spectra Analytics who developed the PATCHS online consultation system and is a shareholder in the company. The remaining authors declare no conflict of interest.

## ETHICS STATEMENT

Ethical approval for this study was granted by the East of Scotland Research Ethics Service (REC reference: 22/ES/0011). Informed consent, written or verbal (if non‐English language), was obtained for all participants in this study.

## Supporting information

Supporting information.

## Data Availability

The data that support the findings of this study are available on request from the corresponding author. The data are not publicly available due to privacy or ethical restrictions.
