# Supplementary material for: Exploring the experiences and preferences of South Asian patients' of primary care in England since COVID‐19
Source: Health Expect. 2024 Feb 5;27(1):e13982. doi: 10.1111/hex.13982 (PMC10844758; doi:10.1111/hex.13982)
Supplement: Supplementary file 1 — Supporting information. [file HEX-27-e13982-s001.docx]

**Interviews and Focus Group Topic Guide: Patients and/ or Carers**

1. **Introduction**

Our research team is looking at the experiences and perceptions of people such as yourself, and/or carers as well as those working in general practice when you have been trying to make appointment and have appointments since COVID-19. We are really interested in finding out more about what people think about the process of getting an appointment since COVID-19, what types of appointments you’ve had with your GP and what you think of having telephone appointments and/or using online systems.

As researchers we are not for or against any particular way of making and conducting appointments, but we would like to understand more about what people really think. We are interested in how people from various backgrounds and their carers are finding booking and having their appointments since COVID-19. Just to note, during the interview we won’t ask you to share details about the reasons why you have booked appointments – you may prefer to keep this private –the focus will be on your views about the process and outcomes of getting and having appointments at your practice since COVID-19.

- Recap details of participation
- Take consent
- Encourage completion of demographic form

1. **Background**

- Ask each participant to introduce themselves
- Consider only using their first name

**Can you tell me about your experiences of using the appointment system at your surgery BEFORE COVID-19**

Prompts:

- ***How*** did you make the appointment request (telephone or online)?
  - Was there more than one way to make the appointment request (Telephone or online)?
    - If so, why did you choose the one you did?
- What happened after you made the appointment request?
- What happened next (Probe follow-up phone call, video-call, face to face (in the surgery) appointment or another outcome and with who?)
- How did you feel about that outcome? (appropriate to get a telephone call? GP vs nurse etc)
- How did you feel about using the triage appointment system your practice has??
  - Why do you feel that way?

**Since COVID-19**

- Did you notice a change in how your practice was working in terms of making an appointment and doing their consultations?
- Are you able to make an appointment in more than one way now vs before (online now)?
- Have you ever been offered or used a video consultation?
  - If so, did you experience technical difficulties (sound quality, picture quality et)
  - Have these issues been addressed?
- Did you prefer this vs telephone? If so, why?
- Did these approaches enable the use of translators if you needed one as a patient or if translating on behalf of a family member ? Which is better for the use of translators – phone or online?
- *DO you feel you have enough knowledge/skills to use your GP’s online services??*
- What does a face-to-face appointment offer vs telephone appointment?
- What does a face-to-face appointment offer vs a video appointment?
  - When would a telephone appointment be seen as appropriate for you?
- Have you faced any difficulties with communicating during the appointment making stage? (e.g. being understood, accessing an interpreter, non-verbal communication needs)
  - if so, have you given any feedback to your practice about these issues??
  - Have you ever not contacted the surgery due to fear of not being understood due to language or any other barriers?
    - Did you go to a walk-in, A&E, instead? Probe: outcome of this.
- People have talked about SA cultural factors and differences that need to be thought about with these newer ways of accessing general practice - could you tell me what these are in your own words?

**Have there been previous occasions where the appointment system *has* worked well for you -could you tell me about them?**

Prompts:

- - What happened?
  - Who did you speak to? (Probe use of translators)
  - How was your appointment request dealt with?
  - Did you speak or see a GP that you knew?
    - Did they speak your preferred language?
  - What made it a good outcome?
  - Did you give any positive feedback?

**Have there been occasions where the appointment system has not worked well for you – could you tell me about them?**

Prompts:

- - What happened?
  - Who did you speak to? (Probe receptionists attitude around culture and use of translators)
  - How was appointment request dealt with?
  - Did you speak or see a GP that you knew?
    - Did they speak your preferred language?
  - What made it an unsatisfactory outcome?
  - Did you complain?

**Are there things you don’t like about the appointment system which you would like to change?**

**Has the appointment system changed how you use the surgery?**

Prompts:

- Is it quicker to get an appointment with the surgery?
- Has it changed how often you go to the surgery?
- Do you see the staff at the surgery less/more?
- Do you like the surgery appointment system?

**Have you had any opportunity to tell the surgery what you think of the appointment system?**

- Have you ever received a feedback text question? Probe: no reply due to language barrier, other cultural considerations.

**Is there anything else about the appointment system at your surgery, which we have not talked about, but you would like to mention?**

Thank Participants
